# Analysis of surgeries for Degenerative lumbarstenosis in elderly patients

**DOI:** 10.12669/pjms.321.8917

**Published:** 2016

**Authors:** Bin Bai, Yuxin Li

**Affiliations:** 1 Bin Bai, MD, Department of Orthopaedics, Tianjin 4th Centre Hospital, Tianjin, 300140, China; 2 Yuxin Li, MD, Department of Nursing, Tianjin 4th Centre Hospital, Tianjin, 300140, China

**Keywords:** Degenerative lumbar stenosis, elderly patients, Surgery, Japanese Orthopedics Association score (JOA), Oswestry disability index (ODI)

## Abstract

**Objective:**

To analyze the effect of decompression alone and combined decompression, fusion and internal fixation procedure for degenerative lumbar stenosis in elderly patients.

**Methods:**

We reviewed 168 lumbar stenosis patients treated using decompression alone or with combined procedures in the department of orthopaedics of Tianjin 4^th^ Centre Hospital from October 2010 to January 2014. The clinical data including age, gender, procedure type, operation time, follow-up period, blood loss, preoperative and postoperative JOA and ODI scores were recorded. The patients were divided into decompression alone group and combined surgeries group according to the procedure type.

**Results:**

The combined surgeries group presented with larger blood loss (p<0.05) and more operation time (p<0.05), compared with the group of decompression alone. The preoperative and postoperative JOA scores were significantly higher (p<0.05), and the ODI scores significantly lower in the decompression alone group (P<0.05), but at the final follow-up, there were no significant difference between the two groups (p>0.05). The complication rate was lower in the group of decompression alone, but there was no significant difference between the two groups (p>0.05).

**Conclusion:**

Both the decompression alone and combined surgeries can result in a satisfactory effects in elderly patients with degenerative lumbar spinal stenosis, but the combined surgeries presented with a relatively higher complication rate.

## INTRODUCTION

Lumbar spinal stenosis (LSS), characterized by anatomic narrowing of spinal canal and leading to dural sac and nerve root compression, is the most frequent disorder inelderly patients.^1^ Although most physicians advocates the disorder should be treated using conservative methods, surgical treatment is indicated in those with LSS who have persistent debilitating symptoms, especially failed conservative treatments.^1^ In addition, many authors suggestthat surgical treatment results in a better outcome than conservative treatment.^2^,^3^

In terms of surgical treatment, the standard procedure for lumbar spinal stenosis is open surgical decompression.^4^ At the same time, with the advance of lumbar spacers and interbody fusion in the field of spine surgery, decompression, interbody fusion and internal fixation have been carried out together for elderly patients with LSS. Current prevailing approaches for LSS include laminectomy, minimally laminectomy in combination with pedicle screw fixation and fusion, and laminectomy with instrumentation.^5^

In a clinical study,^4^ thirty-six patients had the Aperius implant, thirty-five underwent open decompression and both groups followed prospectively, Postacchini concluded lumbar stenosis is unclear,the decompression procedures ensured high chances of good results, and the implant may be indicated for selected patients with moderate stenosis. In addition, many cases with lumbar stenosis have suffered from procedures of posterior decompression, posterior lumbar interbody fusion and pedicle screw fixation. Obviously, the procedures may result in more intraoperative blood loss, higher hospital cost, longer hospital stay and operation time. However, whether the combined procedures present a better outcome than decompression alone for patients with lumbar stenosis is unclear, and the additional value of decompression and arthrodesis compared to decompression alone is debateable,^6^ but few studies have been carried out to clarify the issues.

The objective of the study is to analyze the effect of decompression alone and combined decompression, fusion and internal fixation procedure for degenerative lumbar stenosis in old patients.

## METHODS

In this retrospective study, we collected data for 168 patients suffering from low back pain with intermittent claudication due to degenerative lumbar stenosis. These patients underwent decompression alone or combined decompression, fusion and internal fixation surgeries in our hospital between October 2010 and January 2014. The inclusion criteria included a patient with a primary diagnosis of degenerative lumbar stenosis, whose age ≥60 years, who was treated surgically and the clinical data was integrative. The patients with Paget’s disease, severe osteoporosis or metastasis to the vertebrae, and severe comorbidity that increase the risk to the patient or interfere with the assessment of the study such as severe ischemic heart disease, musculoskeletal or neurological conditions impairing walking ability, cognitive impairment as well as previous lumbar surgeries were excluded.^7^ This study was approved by the institutional review board of our hospital.

In terms of patients with central canal or lateral recess stenosis only, the vertebral lamina fenestration or enlarging lateral recess surgery was carried out. When both central canal and lateral recess stenosis available, then the total laminectomy was performed to enlarge spinal canal. When the preoperative instability is available or excision of facet joint was more than a half, the posterior inter body fusion and pedicle screw fixation was carried out to avoid postoperative instability.

The clinical data, such as age, gender, procedure type, operation time, follow-up period, blood loss and stenosis were recorded for all the included patients. The patients were divided into two groups of decompression alone and combined procedures according to the procedure type. In terms of the evaluation of clinical outcomes, Japanese Orthopedics Association score (JOA)^8^ and Oswestry disability index (ODI)^9^ were used for both groups. The JOA and ODI evaluation were carried out preoperatively, postoperatively and at the final follow-up.

We used SPSS version 21.0 (SPSS Inc., Chicago, IL, USA) for statistical analysis and p < 0.05 was considered significant. Independent 2-sample t test was performed to compare the difference of measurement data, and a chi-square test was used to compare the difference of enumeration data between two groups.

## RESULTS

In the current study, 168 patients were included, 88 were female and 80 male, the average age was 68.6 years and it ranged from 60 to 83 years. Out of 168 cases, the decompression alone was carried out in 110 cases, and the combined decompression, fusion and internal fixation surgery were performed in 58 cases. Thus, the decompression group consisted of 110 patients and the combined surgeries group consisted of 58 cases. The clinical data of the two groups are listed in Table-I. There was no significant difference in gender, age, follow-up period, stenosis level between the two groups (p>0.05).

**Table-I:** The clinical data of two groups in the current study.

	Decompression alone	Combined surgery	Pvalues
Case number	110	58	-
Gender (M/F)	49/61	31/27	P>0.05
Age (year)	67.2±5.4	69.6±6.3	P>0.05
Stenosis level			P>0.05
1 level	55	31	
2 levels	42	19	
3 levels	11	7	
4 levels	2	1	
Follow-up (month)	21.3±4.5	23.1±3.9	P>0.05
Operation time (minute)	85±18	186±32	P<0.05
Blood loss	112±26	348±78	P<0.05

The operation time in the decompression group was 85±18 minutes, in the combined surgeries group was 186 ±32 minutes; the blood loss of operation was 112±26 ml in the decompression group and 348±78 ml in the combined surgeries group. There was significant difference in blood loss (p<0.05) and operation time (p<0.05) between the two groups.

The JOA and ODI evaluation are listed in Table-II. The preoperative and postoperative JOA scores were significantly higher in the decompression group than those in the combined surgeries group (p<0.05), and the ODI scores were significantly lower in the decompression group (P<0.05), but at the final follow-up, there were no significant difference between the two groups (p>0.05). In addition, the postoperative JOA scores were significantly higher in both groups than preoperative scores (p<0.05) and the postoperative ODI scores were significantly lower in both groups than the preoperative scores (p<0.05).

**Table-II:** The comparison of ODI and JOA between the groups.

	Oswestry disability index scores(ODI)		Japanese Orthopedics Association scores (JOA)	

	Decompression alone	Combined surgery	Decompression alone	Combined surgery
Before surgery	54.6±6.7	60.3±6.9	19.5±2.6	14.8±2.4
After surgery	48.5±3.4	54.4±5.7	26.8±3.1	22.6±2.9
Final follow-up	45.2±2.7	47.1±3.1	27.7±3.2	26.8±3.0

In terms of the complications, four patients suffered from dural tear, three patients incision infection in the decompression group and the complication rate was 6.4%. In the combined surgeries group, three patients suffered from dural tear, four patients from incision infection and one patient from cognitive disorder and the complication rate was 13.8%. There was no significant difference in the complication rate between the two groups (p>0.05).

## DISCUSSION

In the current study, we reviewed retrospectively 168 patients older than 60 years with LSS treated surgically in our hospital, to compare the effect of decompression alone and combined surgeries. The study may help surgeons better understand the treatment strategies of LSS in elderly patients.

With the development of medical imaging technology, especially CT and MRI, surgeons can learn the detailed conditions of lumbar spine including the stenosis level, stenosis severity, related lumbar stability together with the symptoms and signs of patients, and subsequently they can determine the treatment strategies before surgery. Complete decompression is critical for patients with LSS in improving clinical symptoms.^10^ However, in some cases, complete decompression may result in lumbar instability, as a result, internal fixation and fusion may need to be carried out to maintain the stability of lumbar spine. Obviously, the combined decompression, fusion and internal fixation may increase the operation time, blood loss and related risk during surgery.

In the study, decompression alone surgery was performed in 110 patients, while combined surgeries were carried out in 58 cases. We found the average operation time and blood loss in the combined surgeries group were significantly higher than those in the decompression alone group, which confirmed the above mentioned viewpoints. At the same time, we found in the combined surgeries group, the complication rate was higher, although no significant difference was available. At this point, we attributed it to the relatively small sample size. We believe if the sample size was increased, the difference may be significant.

We used JOA and ODI to evaluate the effect of treatment in two groups, before and after surgery, the JOA and ODI scores in the decompression alone group were better than those in the combined surgeries group. The spinal stenosis in the combined surgeries group is usually more serious than that in the decompression group, not only central stenosis but also lateral recess stenosis may be available, resulting in more serious symptoms, lower JOA and higher ODI scores in the combined surgeries group before surgery. Also, the combined surgeries need more operation time, and may result in more blood loss, more surgical trauma and higher complications rate, in which the patients may need more time to recover from the surgery. Subsequently, the postoperative evaluation of JOA and ODI is usually not satisfactory as that in the decompression group. However, at the final follow-up, there was no significant difference in JOA and ODI scores between the two groups, demonstrating that both the decompression alone and combined surgeries group can result in a satisfactory outcomes. But, Resnick held a different viewpoint and suggested that internal fixation and fusion could result in a better clinical outcomes than decompression alone.^11^ On the contrary, in a study of seventy-six patients who had symptomatic spinal stenosis, Fischgrund found the use of pedicle screws may lead to a higher fusion rate, but clinical outcome shows no improvement in pain in the back and lower limb, i.e. internal fixation have no significant influence on the clinical outcomes.^12^ In a systematic review and meta-analysis, Machado also concluded that decompression plus fusion is not more effective than decompression alone.^13^ These studies presented with similar points as the current study.

In addition, before surgery three patients in the decompression alone group was diagnosed with degenerative lumbar spondylolisthesis, but at the final follow-up, they also presented with a significant improvement in JOA and ODI scores. In this regards, controversial viewpoints is available. Some author advocated that the degenerative lumbar spondylolisthesis was relatively stable, and limited decompression may not lead to spinal instability. Consequently, these patients may not need further fusion and internal fixation^14^; Nevertheless, in a study of 44 patients with degenerative spondylolisthesis and spinal stenosis, Bridwell reported that there was significantly more spondylolisthesis progression in the decompression alone group.^15^ Our outcomes may support the first viewpoint. However, we can’t make a significant conclusion because only three patients were diagnosed with degenerative lumbar spondylolisthesis before surgery.

### Limitations of the study

First, the current study was a retrospective study. A prospective study may be better in observing patients, collecting and analyzing clinical data. Second, the sample size of the current study was small, which may affect the final conclusion drawn correctly, and a large scale clinical study may be needed in order to obtain a more definite conclusion. Despite the limitations, we have concluded that both the decompression alone and combined surgeries can result in a satisfactory effects in elderly patients with lumbar spinal stenosis.

## References

[1] Hsieh PC (2013). Efficacy of surgical treatment for lumbar stenosis with the X-STOP: an issue in need of closer inspection. World Neurosurg.

[2] Li YJ, Xu HZ, Chen BL, Ning FP (2011). Comparative study of the efficacy of non-surgical and surgical therapy for degenerative lumbar spinal stenosis. Nan Fang Yi Ke Da Xue Xue Bao.

[3] Atlas SJ, Keller RB, Wu YA, Deyo RA, Singer DE (2005). Long-term outcomes of surgical and nonsurgical management of lumbar spinal stenosis:8 to 10 year results from the maine lumbar spine study. Spine (Phila Pa 1976).

[4] Postacchini R, Ferrari E, Cinotti G, Menchetti PP, Postacchini F (2011). Aperius interspinous implant versus open surgical decompression in lumbar spinal stenosis. Spine J.

[5] Zhang L, Miao HX, Wang Y, Chen AF, Zhang T, Liu XG (2015). Limited unilateral decompression and pedicle screw fixation with fusion for lumbar spinal stenosis with unilateral radiculopathy: A retrospective analysis of 25 cases. J Korean Neurosurg Soc.

[6] Burgstaller JM, Porchet F, Steurer J, Wertli MM (2015). Arguments for the choice of surgical treatments in patients with lumbar spinal stenosis - a systematic appraisal of randomized controlled trials. BMC Musculoskelet Disord.

[7] Shang Y, Chen GM, Tang SJ (2015). Therapeutic effect of tuina combined with Jin Gui Shen Qi Decoction on lumbar spinal stenosis. JAcupuncture Tuina Sci.

[8] Hioki A, Miyamoto K, Hosoe H, Sugiyama S, Suzuki N, Shimizu K (2011). Cantilever transforaminal lumbar interbody fusion for upper lumbar degenerative diseases (minimum 2 years follow up). Yonsei Med J.

[9] Zhang Y, Tang S, Chen G, Liu Y (2015). Chinese massage combined with core stability exercises for nonspecific low back pain: a randomized controlled trial. Complement Ther Med.

[10] Hansraj KK, O'Leary PF, Cammisa FP, Hall JC, Fras CI, Cohen MS, Dorey FJ (2001). Decompression, fusion, and instrumentation surgery for complex lumbar spinal stenosis. Clin Orthop Relat Res.

[11] Resnick DK, Choudhri TF, Dailey AT, Groff MW, Khoo L, Matz PG (2005). Guidelines for the performance of fusion procedures for degenerative disease of the lumbar spine. Part 9: fusion in patients with stenosis and spondylolisthesis. J Neurosurg Spine.

[12] Fischgrund JS, Mackay M, Herkowitz HN, Brower R, Montgomery DM, Kurz LT (1997). 1997 Volvo Award winner in clinical studies Degenerative lumbar spondylolisthesis with spinal stenosis: a prospective, randomized study comparing decompressive laminectomy and arthrodesis with and without spinal instrumentation. Spine (Phila Pa 1976).

[13] Machado GC, Ferreira PH, Harris IA, Pinheiro MB, Koes BW, van Tulder M (2015). Effectiveness of surgery for lumbar spinal stenosis: a systematic review and meta-analysis. PLoS One.

[14] He Q, Ruan D, Li H, Zhang C D W (2011). Selection of surgical treatment and analysis of clinical efficacy in degenerative lumbar spinal stenosis. Orthop J China.

[15] Bridwell KH1, Sedgewick TA, O'Brien MF, Lenke LG, Baldus C (1993). The role of fusion and instrumentation in the treatment of degenerative spondylolisthesis with spinal stenosis. J Spinal Disord.

